# Managerial factors affecting milking-abilities of Holstein cattle under intensive production system in Egypt

**DOI:** 10.1007/s11250-024-04271-w

**Published:** 2025-01-11

**Authors:** E. Faid-Allah, Reem S. Mourad, E. I. Saddick, E. Eldahshan

**Affiliations:** Department of Animal Production, Faculty of Agriculture, Menoufia University, Shibin Al Kawm, Egypt

**Keywords:** Milking-ability, Herds’ men, Milking times, Mastitis infection times, Holstein cattle

## Abstract

This article aims to explore milking-ability criteria of Holstein dairy cattle under intensive production system in Egypt and investigate some managerial factors that influence them in dairy farms. The data obtained from five herds belong to a commercial intensive production system farm, Egypt. Data included 3509 records. The values of mean ± SD (CV%) of the milking-ability criteria for Holstein dairy cattle as follow: daily milk yield, kg (dmy), milking duration, min (MD), peak flow rate, kg min^−1^ (PFR), average flow rate, kg min^−1^ (AFR), AFR^0:15^ s of milking, AFR^15:30^ s of milking, AFR^30:60^ s of milking, and AFR^60:120^ s of milking were 30.34 ± 6.76 kg, (22.27%); 4.16 ± 0.79 min (19.06%); 3.62 ± 0.79 (21.77%); 0.28 ± 0.11 (39.48%); 1.40 ± 0.49 (36.37%); 1.26 ± 0.50 (39.95%); and 1.96 ± 0.71 (36.05%), kg min^−1^, respectively. The milking-time (a.m./p.m.) and the herds’ men influenced significantly (*P* ≤ 0.01) on dMY, and milking-ability criteria. In addition, there was a significant effect (*P*˃0.05) of cows’ dry-period on dMY, MD, PFR and AFR. Also, it was clearly appearing that there was a dramatic significant decrease in daily milk yield and AFR by increase mastitis infection times. Milking-ability criteria in Holstien cattle may be go better via good management, hygiene, and well-trained herds’ men. The cubic models are higher in determination coefficient (*p* ≤ 0.05) than linear models for the curve estimates through the time of lactation period for studied traits. The curve of milk yield and milking-abilities were inconstant all over lactation period.

## Introduction

Holstein dairy cattle are known for their high milk production and are widely used in the dairy industry. The milking-abilities of Holstein are influenced by various factors, including management practices. Additionally, proper care is crucial for optimizing the milking-ability of Holstein dairy cattle. A good milking procedure reduces stress for cows and milkers, leading to a more efficient and productive milking process. In recent decades, cow’s milking-abilities have become increasingly important as milk yield criteria for dairy producers around the world to reduce labor-time, minimize total milking time, and increase cow and herd overall welfare, without affecting the milk yield (Samoré et al. 2011). Milking-ability traits includes milk duration (MD), peak flow rate (PFR), and average flow rates (AFR) (Lee and Choudhary 2006). Increasing milking speed makes reduction in milking working-time, this leads to decrease the cost of labor and operating energy. Also, weariness of milking equipment (Vosman et al. 2014). Profitability of milk production depends on the cow’s milking efficiency, that affected by many factors especially milking frequency, milk yield, and milking speed (Aerts et al. 2022).

The time of milking (a.m./p.m.) significantly affected milk yield (Adebosin et al. 2010; Garantjang et al. 2020; Roshanfekr et al. 2010) and significantly affected milk flow rate (Roshanfekr et al. 2010). On the other hand, the difference between morning and evening milking was not significant for milk yield. However, milk flow rate was significant (Anand et al. 2010). Herd's men (milkers) have a great influence on milk production and milking ability. Before calving, a dry period is required for cows in order to regenerate the mammary gland (renewal of udder tissue), both at physical and physiological levels (Kuhn et al. 2005).

Dairy cows need a certain dry period between two lactations to give a regular and enough milk in the second and later lactations. This dry period is associated with dairy cows' milk yield, milk composition, reproductive performance. Most dairy farms have a dry-period of 51–60 days (Kiyici et al. 2020). At least 40 days of dry-period is optimal, while an extended dry-period over 80 days could lead to a negative impact on milk production and animal welfare (Radu et al. 2021). A dry-period < 40 days or > 70:80 days was associated with reduced milk yield and amount of chemical components in the subsequent lactation (Annen et al. 2004; Kuhn et al. 2007; Sawa et al. 2012).

Bovine mastitis, an inflammation of the mammary gland, is the most common disease of dairy cattle causing economic losses due to reduced yield and poor quality of milk (Cheng and Han 2020; Gomes and Henriques 2016). Also, mastitis cows often never recovered their potential yield (Gröhn et al. 2004). The accurate description of lactation curves is an interesting topic for breeding and management purposes in dairy farming. Scientists have proposed several mathematical functions to mimic milk production records over time (Pollott 2000), as follow: log-quadratic, LQ (Adediran et al. 2012); modified gamma, MG (Morant and Gnanasakthy 1989); modified incomplete gamma (Wood 1967); modified LQ, LQn; modified MG, MGn; incomplete gamma; exponential (Wilmink 1987); quadratic polynomial (Dave 1971); parabolic exponential (Sikka 1950); bicompartmental (Ferguson and Boston 1993); Dijkstra (Dijkstra et al. 1997); Pollott (Pollott and Gootwine 2000); modified Pollott; Legendre polynomial (Kirkpatrick et al. 1994); cubic splines (Green and Silverman 1993). This article aims to explore milking-ability criteria of Holstein dairy cattle in Egypt and investigate some factors that influence them in dairy farms.

## Materials and methods

### Data

The data collected from 5 herds at Dina farm, a commercial farm specializing in agriculture investment, located on the Cairo-Alex., desert road ≈80-km. The dataset consists of information on 3509 records that presented the period from 2015 to 2022. Various managerial factors were considered, the times of milking, the herds’ men (milking team), dry-period and mastitis infection times. Delaval-Alpro management system and Dairy comp-305 (DC-305) were used as the computer record keeping system.

### Management and feeding system

Animals were fed ad-libitum using the TMR system as recommended by National Research Center (NRC 2001). Dairy cows were fed 3 rations according to their production and stage of lactation as fresh, high, and low. Fresh rations were fed to the cow from 0:25 days in milk then cows were fed high milking ration until days in milk increases and the average daily milk production decreases to < 20 kg-milk day^−1^, and then it’s fed on a low-ration production to dry-off. The rations were formulated of corn silage, alfalfa-hay, soybean meal, corn kernels, wheat bran, corn gluten feed, protected-fats, and vitamin and mineral-mixtures.

The Holstein cattle were housed in outdoor shaded pens and grouped according to productive and reproductive status. Artificial insemination was applied using frozen semen of Holstein sire. Pregnancy was confirmed by rectal palpation after 35 days of AI.

The cows were milked thrice daily at 3 a.m., 11 a.m., and 7 p.m. each round takes ≈10 min by Delaval milking-parlors. Cows identified in the milking-parlor with e-chipset. The milking order is as follows: Fresh cows enter the milking-parlor as 1st-group, then 1st-parity in 2nd-group, after that ≥ 2nd-parity in 3rd-group and finally, low productivity cows in 4th-group. The cows were taken from the shelter to the gathering area of ​​the milking-parlor to begin the next routine procedure as follow: Soaking all teats in a pre-bath as a disinfectant before milking, then striping all nipples, then drying it with a tissue; after that, attaching the milking cluster. Once the cow had been milked, all teats were soaked in post-milking disinfectant.

### Studied criteria

Studied traits of milking-abilities were daily milk yield (dMY), kg, milking duration (MD), min, peak flow rate (PFR), kg min^−1^, average flow rate (AFR), kg min^−1^ and average flow rate during milking intervals as follow: AFR^0:15^ s of milking, AFR^15:30^ s of milking, AFR^30:60^ s of milking, AFR^60:120^ s of milking.

The milking-ability variables described as follow: Milking duration was the actual time taken to milk out a cow as a time span between time any teat started giving milk until last teat stopped giving milk (Gäde et al. 2006); Peak flow rate was maximum milk harvested during a one min interval. (Petersen et al. 1986); Average flow rate was milk harvested until machine stripping divided by cumulative time (Petersen et al. 1986). These criteria are automatically measured during milking via Delaval-Alpro management system and recorded by Dairy comp-305 (DC-305).

### Statistical analysis

Descriptive statistics including means ± standard deviation (SD); Mode as the most frequent value; first quartile (1st Quartile) is the value that represent more than 25% of variables data; second quartile (2nd Quartile) that represent more than 50% of variables data (2nd Quartile); third quartile (3rd Quartile) that represent more than 75% of variables data and coefficient of variation (CV%). The lactation curve models were analyzed by linear and cubic models. In addition, factors affecting studied criteria were analyzed by general linear model (GLM) and Duncan test at α = 0.05 as a following model:

$${Y}_{ijklm}=\mu + {p}_{i}+{t}_{j}+{o}_{k}+{h}_{l}+{e}_{ijklm}$$

where: Y_ijklm_ = The individual observation; μ = Overall mean; p_i_ = Fixed effect of i^th^ times of milking, i = 1:3 (1, 1st-milking at 3 a.m.; 2, 2nd-milking at 11 a.m.;3, 3rd-milking at 7 p.m.); t_j_ = Fixed effect of j^th^ herds’ men, j = 1:5; o_k_ = Fixed effect of k^th^ categories of dry-period, k = 1:3 (1, ≤ 59 days; 2, 60–90 days; 3, ≥ 91 days). H_l_ = Fixed effect of l^th^ times of mastitis infections, l = 0:4 (0, 1, 2, 3, & ≥ 4 times); e_ijklm_ = Error term NID (0,1).

Descriptive statistics, the lactation curve models and general linear model were analyzed via Statistical Analysis System computer program (SAS 2002).

## Results

### Statistical description

Milking-abilities mainly includes some criteria such as milking duration that refers to the time taken to complete the milking process for an individual cow. In addition. peak flow that refers to the maximum rate at which milk is expelled from the udder during milking. It is an important indicator of udder health and milk production efficiency. Also, Average flow rate refers to the average speed at which milk is released during the entire milking process. This parameter reflects the overall efficiency of milk extraction.

Table 1 displays the mean ± SD (CV%) of the milking-ability traits for Holstein dairy cattle as follow: daily Milk yield, milking duration, peak milk flow rate, average flow rate, AFR^0:15^, AFR^15:30^, AFR^30:60^, and AFR^60:120^ were 30.34 ± 6.76 kg, (22.27%); 4.16 ± 0.79 min (19.06%); 3.62 ± 0.79 (21.77%); 1.76 ± 0.39 (22.52%); 0.28 ± 0.11 (39.48%); 1.40 ± 0.49 (36.37%); 1.26 ± 0.50 (39.95%), and 1.96 ± 0.71 (36.05%) kg min^−1^, respectively.

**Table 1:** The milking-ability traits for Holstein dairy cattle

Milking-ability traits	Mean ± SD	Mode	1^st^ Quartile	2^nd^ Quartile	3^rd^ Quartile	CV, %
Daily Milk yield, kg	30.34 ± 6.76	32	26.50	30.10	34.19	22.27
Milking duration, min	4.16 ± 0.79	4.46	3.62	4.08	4.62	19.06
Peak milk flow rate, kg min^−1^	3.62 ± 0.79	3.60	3.08	3.61	4.13	21.77
Milk flow rate (kg min^−1^)						
Average flow rate, AFR	1.76 ± 0.39	1.71	1.49	1.84	2.01	22.52
AFR^0:15^	0.28 ± 0.11	0.17	0.20	0.25	0.33	39.48
AFR^15:30^	1.40 ± 0.49	1.23	1.00	1.30	1.64	36.37
AFR^30:60^	1.26 ± 0.50	1.11	0.90	1.19	1.56	39.95
AFR^60:120^	1.96 ± 0.71	1.80	1.45	1.89	2.39	36.05

*dMY* daily Milk yield, kg, *MD* milking duration, min, *PFR* peak milk flow rate, kg min^−1^, *AFR* average flow rate, kg min^−1^, *AFR*^*0:15*^ 0:15, seconds of milking, kg min^−1^, *AFR*^*15:30*^ 15:30, seconds of milking, kg min^−1^, *AFR*^*30:60*^ 30:60, seconds of milking, kg min^−1^, *AFR*^*60:120*^ 60:120, seconds of milking kg min^−1^, *Mean* ± *SD* mean ± standard deviation, *Mode* most frequent value, *1st Quartile* first quartile is the value that represent more than 25% of variables data, *2nd Quartile* second quartile that represent more than 50% of variables data, *3rd Quartile* third quartile that represent more than 75% of variables data, *CV (%)* coefficient of variation

### Managerial Factors affecting Milking-abilities

A) Milking-times
  Table 2 shows milk yield and milking-ability traits of Holstein dairy cattle at various times of milking. It was clearly appearing that the cattle produced the most milk yield (8.86 kg) during 2nd-milking at 11 a.m. followed by 8.60 kg during 1st-milking at 3 a.m., the lowest milk yield (8.37 kg) was recorded at 3rd-milking at 7 p.m. Among the various times of milking, the 3rd-milking at 7 p.m. recorded the longest milking duration (4.22 min), while the shortest was attained at the 1st-milking at 3 a.m. (4.10 min), the 2nd-milking had intermediate value 4.17 min It is interesting to note that most milk yield during 2nd-milking was associated with the highest Peak milk flow rate, (3.69 kg min^−1^) followed by 3.60, and 3.57 kg min^−1^, at 3rd, and 1st-milking, respectively. These differences were highly significant (*p* ≤ 0.01).

**Table 2:** Effect of milking-times on milking-ability traits of Holstein dairy cattle

Milking-ability criteria (LSM ± SD)	Times of milking (№ = 3219)		
	1st milking, 3 a.m	2nd milking, 11 a.m	3rd milking, 7 p.m
Milk yield per time, kg *(P* ≤ *0.001)*	8.60^b^ ± 1.69	8.86^a^ ± 1.75	8.37^c^ ± 1.66
Milking duration, min *(P* ≤ *0.001)*	4.10^c^ ± 0.80	4.17^b^ ± 0.84	4.22^a^ ± 0.83
Peak milk flow rate, kg min^−1^ *(P* ≤ *0.001)*	3.57^b^ ± 0.84	3.69^a^ ± 0.87	3.60^b^ ± 0.86
Milk flow rate, kg min^−1^			
Average flow rate, AFR *(P* ≤ *0.001)*	1.72^c^ ± 0.39	1.81^a^ ± 0.44	1.76^b^ ± 0.44
AFR^0:15^ *(P* ≤ *0.001)*	0.27^b^ ± 0.12	0.29^a^ ± 0.13	0.27^b^ ± 0.12
AFR^15:30^ *(P* ≤ *0.001)*	1.30^b^ ± 0.53	1.46^a^ ± 0.54	1.32^b^ ± 0.53
AFR^30:60^ *(P* ≤ *0.001)*	1.22^b^ ± 0.55	1.36^a^ ± 0.59	1.19^c^ ± 0.52
AFR^60:120^ *(P* ≤ *0.001)*	1.97^b^ ± 0.76	2.05^a^ ± 0.78	1.87^c^ ± 0.74

Means within column classification followed by different superscript are different significantly (*P* ≤ 0.05)
  From the same table it is clearly appears that the average of milk flow rate during all over milking (AFR) and its intervals, 0:15, 15:30, 30:60, and 60:120 s of milking, were the greatest during 2nd-milking at 11 a.m. as follows: 1.81, 0.29, 1.46, 1.36, and 2.05 kg min^−1^, respectively (*p* ≤ 0.01); Compared with those recorded during the other times of milking which were almost the same during 1st-milking as follows: 1.72, 0.27, 1.30, 1.22, and 1.97 kg min^−1^ respectively; and during 3rd-milking as follows: 1.76, 0.27, 1.32, 1.19, and 1.87 kg min^−1^ respectively.

B) Herds' men (Milking team)
  Table 3 indicates that the herds’ men had a remarkably significant impact (*P* ≤ 0.01) on dMY, PFR, MD, and AFR. Table 3 reveals that the animals milked by the 1st-milkers’team (1st-team) produced the highest daily milk yield (32.17 kg) followed by the 3rd-team (31.25 kg) then the 4th-team (30.38 kg) then the 2nd-team (30 kg) but the 5th-team was the lowest (27.42 kg). The milking took the longest time (milking duration) by the 2nd-team (4.97 min) and the lowest by the 3rd-team (3.75 min). The rest of the teams had intermediate values as follows: 4.10, 4.22, and 3.83 min, for the 1st, 4th, 5th-team respectively.

**Table 3:** Effect of herds' men on Milking-ability criteria of Holstein dairy cattle

Milking-ability criteria (LSM ± SD)	Herds' men teams				
	1^st^-team (№ = 626)	2^nd^-team (№ = 695)	3^rd^-team (№ = 611)	4^th^-team (№ = 810)	5^th^-team (№ = 594)
Daily Milk yield, kg *(P* ≤ *0.001)*	32.17^a^ ± 6.40	30.00^c^ ± 6.51	31.25^b^ ± 6.87	30.38^c^ ± 7.11	27.42^d^ ± 6.70
Milking duration, Min *(P* ≤ *0.001)*	4.10^c^ ± 0.55	4.79^a^ ± 0.75	3.75^e^ ± 0.65	4.22^b^ ± 0.90	3.83^d^ ± 0.71
Peak milk flow rate, kg min^−1^ *(P* ≤ *0.001)*	3.67^b^ ± 0.67	3.63b^c^ ± 0.71	3.32^d^ ± 0.76	3.56^c^ ± 0.90	3.85^a^ ± 0.86
Milk flow rate, kg min^−1^					
Average flow rate, AFR *(P* ≤ *0.001)*	1.58^d^ ± 0.25	1.58^d^ ± 0.30	1.80^b^ ± 0.41	1.70^c^ ± 0.40	2.15^a^ ± 0.37
AFR^0:15^ *(P* ≤ *0.001)*	0.27^c^ ± 0.07	0.22^d^ ± 0.05	0.38^a^ ± 0.12	0.29^b^ ± 0.11	0.22^d^ ± 0.07
AFR^15:30^ *(P* ≤ *0.001)*	1.33^c^ ± 0.38	1.24^d^ ± 0.36	1.70^a^ ± 0.52	1.39^b^ ± 0.55	1.11^e^ ± 0.40
AFR^30:60^ *(P* ≤ *0.001)*	1.23^c^ ± 0.44	1.32^b^ ± 0.43	1.50^a^ ± 0.55	1.14^d^ ± 0.54	1.14^d^ ± 0.42
AFR^60:120^ *(P* ≤ *0.001)*	1.63^e^ ± 0.47	2.02^c^ ± 0.58	2.40^a^ ± 0.79	1.72^d^ ± 0.71	2.15^b^ ± 0.64

Means within column classification followed by different superscript are different significantly (*P* ≤ 0.05)
  It is interesting to note that the lowest milk yield (27.42 kg) that achieved by 5th-team was associated with the highest Peak milk flow rate, (3.85 kg min^−1^) and the highest AFR (2.15 kg min^−1^). The lowest Peak milk flow rate was recorded with 3rd-team (3.32 kg min^−1^) and the rest of the teams had intermediate values as follows: 3.67, 3.63, and 3.56 min, for the 1st, 2nd, 4th-team, respectively. These differences were highly significant (*p* ≤ 0.01). This effect of milking teams (herd's men) on milk yield and milking abilities may be because on animal's temperament and milking routines.
  The same AFR (1.58 kg min^−1^) were recorded with the 1st,and 2nd-team and were 1.80, and 1.70 kg min^−1^ with the 3rd, and 4th-team. It is interesting to note that high milk yield (31.25 kg) that achieved by 3rd-team was associated with the highest milk flow rates during all studied milking intervals as following: 0.38, 1.70, 1.50, and 2.40 kg min^−1^ for 0:15, 15:30, 30:60, and 60:120 s of milking.

C) Dry Period Categories
  The effect of different dry-periods on Milking-ability criteria of Holstein dairy cattle are listed in Table 4. There was a significant effect (*P*˃0.05) of cows’ dry-period on dMY, MD, PFR, and AFR, where daily milk yields elevated (32.28 kg day^−1^) significantly as 60–90 days of dry before calving. On the other hand, the daily milk yield as ≤ 59 days, and ≥ 91 days dry-period was almost equal as follows: 30.44, and 30.66 kg day^−1^respectively. The lowest milking duration and Peak milk flow rate were recorded as ≥ 91 days of dry-period which were 3.98 min and 3.06 kg min^−1^ The milking took nearly the same time (milking duration) with ≤ 59 days, and 60–90 days of dry-periods as follows: 4.26, and 4.28 min respectively. Also, Peak milk flow rates were nearly the same with ≤ 59 days and 60–90 days of dry-periods as follows: 3.64, and 3.59 kg min^−1^, respectively.

**Table 4:** The effect of different dry-periods on milking-ability criteria of Holstein dairy cattle

Milking-ability criteria (LSM ± SD)	Categories of Dry Period		
	≤ 59 days (№ = 1531)	60–90 days (№ = 1481)	≥ 91 days (№ = 135)
Daily Milk yield, kg *(P* ≤ *0.001)*	30.44^b^ ± 6.73	32.28^a^ ± 6.93	30.66^b^ ± 7.20
Milking duration, min *(P* ≤ *0.001)*	4.26^a^ ± 0.76	4.28^a^ ± 0.81	3.98^b^ ± 0.82
Peak milk flow rate, kg min^−1^ *(P* ≤ *0.001)*	3.64^a^ ± 0.75	3.59^a^ ± 0.78	3.06^b^ ± 0.88
Milk flow rate, kg min^−1^			
Average flow rate, AFR *(P* ≤ *0.001)*	1.71^a^ ± 0.34	1.74^a^ ± 0.38	1.43^b^ ± 0.44
AFR^0:15^ *(P* ≤ *0.001)*	0.28^ab^ ± 0.10	0.29^a^ ± 0.11	0.27^b^ ± 0.09
AFR^15:30^ *(P* = *0.001)*	1.38^a^ ± 0.48	1.43^a^ ± 0.52	1.29^b^ ± 0.50
AFR^30:60^ *(P* = *0.001)*	1.29^a^ ± 0.47	1.34^a^ ± 0.53	1.17^b^ ± 0.54
AFR^60:120^ *(P* ≤ *0.001)*	1.90^a^ ± 0.66	2.00^a^ ± 0.75	1.59^b^ ± 0.66

Means within column classification followed by different superscript are different significantly (*P* ≤ 0.05)

D) Times of Mastitis Infection
  Any udder health issues, such as mastitis or udder infections, can significantly affect milking-abilities for cows. Regular monitoring and prompt treatment of udder health problems are crucial in maintaining optimal milking process.
  Table 5 shows the effect of mastitis infection times on milking-ability criteria of Holstein dairy cattle. It was clearly appearing that There was a dramatic significant decrease in daily milk yield and AFR by increase mastitis infection times, which were 30.9 kg, and 1.81 kg min^−1^ respectively of non- infected animals. While milk yield was recorded 29.52, 29.14, 28.66, and 28.1 kg for infected animals 1, 2, 3, and ≥ 4 times respectively. Also, AFR were 1.72, 1.64, 1.61, and 1.5 kg min^−1^, respectively.

**Table 5:** Effect of mastitis infection times on milking-ability criteria of Holstein dairy cattle

Milking-ability criteria (LSM ± SD)	Times of Mastitis Infection				
	Zero (№ = 2281)	1 (№ = 691)	2 (№ = 280)	3 (№ = 128)	≥ 4 (№ = 126)
Daily Milk yield, kg *(P* ≤ *0.001)*	30.90^a^ ± 7.39	29.52^b^ ± 5.29	29.14^bc^ ± 4.68	28.66^bc^ ± 4.68	28.10^c^ ± 4.62
Milking duration, min *(P* ≤ *0.001)*	4.10^c^ ± 0.87	4.26^b^ ± 0.77	4.34^b^ ± 0.85	4.29^b^ ± 0.77	4.51^a^ ± 0.86
Peak milk flow rate, kg min^−1^ *(P* ≤ *0.001)*	3.65^a^ ± 0.80	3.63^ab^ ± 0.75	3.48^b^ ± 0.75	3.53^ab^ ± 0.78	3.26^c^ ± 0.71
Milk flow rate, kg min^−1^					
Average flow rate, AFR *(P* ≤ *0.001)*	1.81^a^ ± 0.40	1.72^b^ ± 0.36	1.64^c^ ± 0.33	1.61^c^ ± 0.35	1.50^d^ ± 0.30
AFR^0:15^ *(P* = *0.185)*	0.27 ± 0.11	0.28 ± 0.11	0.27 ± 0.10	0.29 ± 0.11	0.27 ± 0.10
AFR^15:30^ *(P* = *0.043)*	1.35^ab^ ± 0.49	1.39^b^ ± 0.50	1.34^b^ ± 0.50	1.46^a^ ± 0.46	1.33^b^ ± 0.45
AFR^30:60^ *(P* = *0.002)*	1.24^b^ ± 0.50	1.28^ab^ ± 0.48	1.31^ab^ ± 0.50	1.37^b^ ± 0.56	1.34^b^ ± 0.47
AFR^60:120^ *(P* ≤ *0.001)*	1.99^a^ ± 0.72	1.93^a^ ± 0.68	1.87^a^ ± 0.63	1.92^a^ ± 0.69	1.73^b^ ± 0.62

Means within column classification followed by different superscript are different significantly (*P* ≤ 0.05)

### The curve estimation for Milking-abilities all over lactation period

**Fig. 1:**
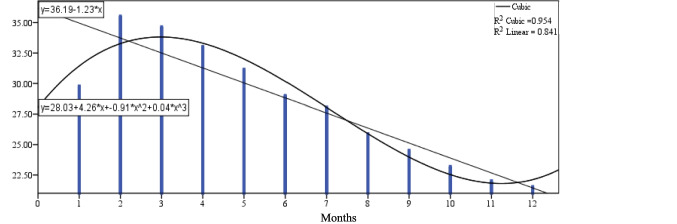
The curve estimation for dMY of Holstein cattle all over lactation period

**Fig. 2:**
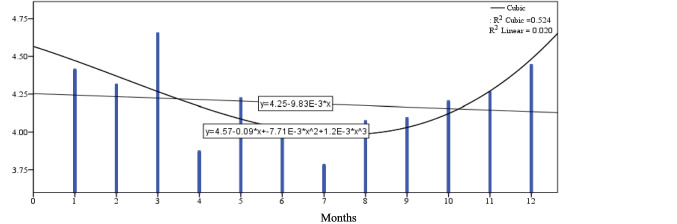
The curve estimation for MD of Holstein cattle all over lactation period

**Fig. 3:**
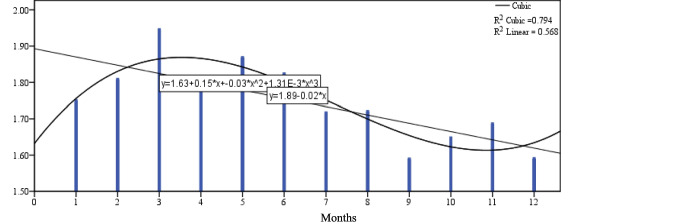
The curve estimation for AFR of Holstein cattle all over lactation period

**Fig. 4:**
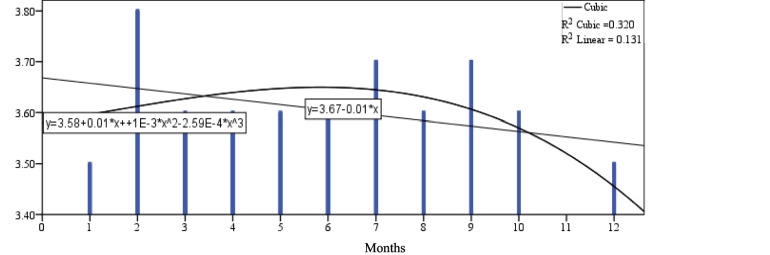
The curve estimation for PFR of Holstein cattle all over lactation period

Figure 1 shows that the daily milk yield (dMY) increases from 1st-month to be the highest value in the 2nd month, then reduces gradually to the end of 12th-months at the end of lactation curve. Figure 2 shows the milk duration (MD) tend to be highest during lactation time at the early stage because of high production of milk with high AFR and at late stages because low AFR with low DMY and gradually decline at the med of lactation as a comparison with two tails of lactation period through 12 months. Figure 3 shows the average flow rate (AFR) tends to be highest during the early stages to get highest value at 3rd month of lactation and gradually decline as lactation progresses. The two curves of AFR, and dMY are similar in shapes at Figs. 1, and 3. Figure 4 shows Peak flow rates (PFR) tend to be highest during the early stages of lactation and gradually decline as lactation progresses.

Table 6 and Figs. 1:4 show that there was a significant difference between the linear models and cubic models (*p* ≤ 0.05) for dMY, MD, AFR and PFR estimates. The cubic model is higher in determination coefficient for the curve estimates through the time for dMY (R^2^_cubic_ = 0.954 vs R^2^_linear_ = 0.841), MD (R^2^_cubic_ = 0.524 vs R^2^_linear_ = 0.020), AFR (R^2^_cubic_ = 0.794 vs R^2^_linear_ = 0.568), PFR (R^2^_cubic_ = 0.320 vs R^2^_linear_ = 0.131).

**Table 6:** The cubic models summary for the curve estimation for milking-abilities all over lactation period

Cubic Model -Coefficients dMY					
Daily milk yield	Unstandardized Coeff		Standardized Coeff	t	Sig
	b	Std. Error	Beta		
(Constant)	28.034	1.986		14.113	0.000
X	4.265	1.270	3.181	3.357	0.010
X^2^	−0.907	0.222	−9.033	−4.075	0.004
X^3^	0.043	0.011	5.069	3.769	0.005
R	R Square	Adj. R Square	Std. Error	F	Sig
0.977	0.954	0.937	1.214	55.435	0.000
Cubic Model -Coefficients MD					
Milking duration	Unstandardized Coeff		Standardized Coeff	t	Sig
	b	Std. Error	Beta		
(Constant)	4.565	0.331		13.812	0.000
X	−0.087	0.211	−1.260	−0.413	0.690
X^2^	−0.008	0.037	−1.486	−0.208	0.840
X^3^	0.001	0.002	2.769	0.639	0.540
R	R Square	Adj. R Square	Std. Error	F	Sig
0.724	0.524	0.346	0.202	2.936	0.099
Cubic Model -Coefficients AFR					
Average flow rate	Unstandardized Coeff		Standardized Coeff	t	Sig
	b	Std. Error	Beta		
(Constant)	1.632	0.095		17.222	0.000
X	0.150	0.061	4.973	2.480	0.038
X^2^	−0.028	0.011	−12.455	−2.655	0.029
X^3^	0.001	0.001	6.907	2.427	0.041
R	R Square	Adj. R Square	Std. Error	F	Sig
0.891	0.794	0.717	0.058	10.308	0.004
Cubic Model -Coefficients PFR					
Peak flow rate	Unstandardized Coeff		Standardized Coeff	t	Sig
	b	Std. Error	Beta		
(Constant)	3.581	0.165		21.665	0.000
X	0.015	0.106	0.510	0.140	0.892
X^2^	0.001	0.019	0.463	0.054	0.958
X^3^	0.000	0.001	−1.429	−0.276	0.790
R	R Square	Adj. R Square	Std. Error	F	Sig
0.565	0.320	0.065	0.101	1.253	0.353

## Discussion

### Statistical description

The values of Table 1 agree with many investigators that studied PFR and AFR for Holstein cows that ranged from 2.94 ± 1.24 kg min^−1^; 2.05 ± 0.74 kg min^−1^ (Cho et al. 2013) to 3.7 ± 1.07 kg min^−1^; 2.34 ± 0.68 kg min^−1^ (Ahn et al. 2005) and tell to 2.49 ± 0.57 kg min^−1^; 1.66 ± 0.36 kg min^−1^ in Italian Brown Swiss (Bobić et al. 2020), respectively. Many investigators represent the milking-ability criteria for many dairy cattle breeds as follow: In Holstein cows, the average of dMY per time of milking was 12.26 ± 4.06 kg time^−1^; AFR was 2.34 ± 0.68 kg m^−1^; PFR was 3.7 ± 1.07 kg m^−1^; MD was 5.33 ± 1.99 min (Ahn et al. 2005); the average of dMY per time of milking was 13.02 ± 4.09 kg; AFR was 2.28 ± 0.51 kg m^−1^; PFR was 3.58 ± 0.79 kg m^−1^; MD was 5.57 ± 1.67 min (An et al. 2006); the average of dMY per time are 13.73 ± 4.28 kg; AFR was 2.3 ± 0.61 kg m^−1^; PFR was 3.28 ± 0.88 kg m^−1^; MD was 6.20 ± 1.99 min (Lee and Choudhary 2006); the average of dMY per day was 26.6 kg; AFR was 2.05 ± 0.74 kg m^−1^; PFR was 2.94 ± 1.24 kg m^−1^; MD was 6.65 ± 2.62 min (Cho et al. 2013). Overall means for AFR, MD and dMY were 1.37 ± 0.004 kg min-1, 16.70 ± 0.017 min and 21.32 ± 0.026 kg for Holstein Cows, respectively (Kaygisiz 2015). The values of AFR and MD were 2 ± 0.0 kg min^−1^ and 360.9 ± 4.7 s in Turkey, respectively (Şahin et al. 2024).

In Italian Holstein, the mean of dMY day^−1^ is 28.8 ± 8.8 kg; PFR is 3.8 ± 1.2 kg m^−1^; MD is 6.9 ± 2.4 min for different parities (Sandrucci et al. 2007); the mean of dMY day^−1^ is 29.03 ± 9.92 kg; AFR is 2.59 ± 0.91 kg m^−1^ for different parities in Polish Holstein (Aerts et al. 2022). In German Holstein, the average of dMY per time of milking is 14.92 ± 4.48 kg; AFR is 4.14 ± 1.16 kg m^−1^; PFR is 5.93 ± 1.47 kg m^−1^; MD is 4.97 ± 1.82 min for > 1st-Parity and AFR is 2.3 ± 0.6 kg m^−1^; PFR is 4 ± 1.3 kg m^−1^; MD is 12.9 ± 4.1 min for different parities (Gäde et al. 2007); the average of dMY is 26.6 ± 7.26 kg; AFR is 2.15 ± 1.27 kg m^−1^ for different parities in German Holstein (Špehar et al. 2017).

Many investigators mentioned that the values of MD ranged from 5.71 to 12.9 min (Povinelli et al. 2003; Carlström et al. 2013; Bobić et al. 2020). Higher PFR values were noted in Swedish Holstein cattle (Carlström et al. 2013) and in Dutch Holstein cattle (Gäde et al. 2007); A slightly lower PFR in jersey cattle (Bobić et al. 2020).

The daily milk yield with test-day record per lactation in Holstien cattle were 8.4, 8.39 and 8.57, kg for 1st, 2nd, and 3rd-lactation, respectively (DeGroot et al. 2007); the daily Milk yield were 18.59 ± 6.63, 20.42 ± 7.67 and 20.53 ± 8.03 kg for the 1st,2nd, and ≥ 3-parity in Tunisia, respectively (Bouallegue et al. 2015). In Italian Holstein, Averages of dMY was 14.9 kg; AFR was 2.47 kg m^−1^; PFR was 3.92 kg m^−1^; MD was 7.17 min for > 1st-Parity and Averages of dMY was 11.85 ± 3.62 kg; AFR was 2.1 ± 0.63 kg m^−1^; PFR was 3.21 ± 1 kg m^−1^; MD was 8.36 ± 2.33 min (Gray et al. 2011).

In Jersey, the average of dMY per time of milking is 13.5, kg; AFR is 2.38, kg m^−1^; PFR is 3.35, kg m^−1^; MD is 6.78, min for 1st-Parity (Sandrucci et al. 2007). In Italian Brown Swiss, the average of dMY is 22.23 ± 8.02 kg; AFR is 1.66 ± 0.36, kg m^−1^; PFR is 2.49 ± 0.57 kg m^−1^; MD is 5.71 ± 2.34 min for different parities (Bobić et al. 2020).

### Managerial factors affecting Milking-abilities

A) Milking-times
  The time of milking (a.m./p.m.) influenced significantly milk yield and rate of milk flow, which were higher in the morning (Roshanfekr et al. 2010); this may be due to the minimum daily temperature and its suitability at morning compared to evening. On the other hand, the difference between morning and evening milking was not significant for milk yield. Although its effect of milk flow rate was significant (Anand et al. 2010).
  The values of Table 2 agree with many investigators who represent the milking-ability criteria at different times of milking (a.m./p.m.) for many dairy cattle breeds as follow: In Holstein, the averages of dMY are 14.14 ± 3.28 kg for 1st-time, and 9.94 ± 2.94 kg for 2nd-time; AFR is 2.42 ± 0.7 kg m^−1^ for 1st-time, and 2.25 ± 0.65 kg m^−1^ for 2nd-time; PFR is 3.76 ± 1.06 kg m^−1^ for 1st-time, and 3.62 ± 1.07 kg m^−1^ for 2nd-time; MD is 5.97 ± 2.06, min for 1st-time, and 4.47 ± 1.54 kg m^−1^ for 2nd-time (Ahn et al. 2005).
  In Swedish Holstein, the averages of dMY are 10.98 ± 3.12 kg for 1st-time, and 12.69 ± 4.04 kg for 2nd, and 3rd-times; AFR is 3.65 ± 1.08, kg m^−1^ for 1st-time, and 4.14 ± 1.16 kg m^−1^ for 2nd & 3rd-times; PFR is 5.32 ± 1.35 kg m^−1^ for 1st-time, and 5.93 ± 1.47 kg m^−1^ for 2nd & 3rd-times; MD is 4.58 ± 1.61 min for 1st-time, and 4.97 ± 1.82 kg m^−1^ for 2nd & 3rd-times (Carlström et al. 2013). The average of milk yield at the morning-milking was higher (8.54 ± 2.78 L day^−1^) than afternoon-milking (5.89 ± 1.89 L day^−1^) in Indonesian cattle herd (Garantjang et al. 2020).
B) Herds' men (Milking team)
  Based to the values of Table 3, It is interesting to note that the lowest milk yield (27.42 kg) that achieved by 5th-team was associated with the highest Peak milk flow rate, (3.85 kg min^−1^) and the highest AFR (2.15 kg min^−1^). The lowest Peak milk flow rate was recorded with 3rd-team (3.32 kg min^−1^) and the rest of the teams had intermediate values as follows: 3.67, 3.63, and 3.56 min, for the 1st, 2nd, 4th-team, respectively. These differences were highly significant (*p* ≤ 0.01). This effect of milking teams (herd's men) on milk yield and milking abilities may be because on animal's temperament and milking routines.
  A decline in milk yield and duration of milking, when a relief milkers milked cows (Lidfors et al. 1999; Knierem 1991). In addition, there was no effect on milk yield when the aversive handler was present at milking. Also, there were no differences in duration of milking according to which handler were present (Munksgaard et al. 2001).
  The same AFR (1.58 kg min^−1^) were recorded with the 1st,and 2nd-team and were 1.80, and 1.70 kg min^−1^ with the 3rd, and 4th-team. It is interesting to note that high milk yield (31.25 kg) that achieved by 3rd-team was associated with the highest milk flow rates during all studied milking intervals as following: 0.38, 1.70, 1.50, and 2.40 kg min^−1^ for 0:15, 15:30, 30:60, and 60:120 s of milking. This may be due to good pre-stimulation routines and gentle handling by the 3rd-team. Pre-stimulation positively influenced the parameters of milk flow (Tancin et al. 2007).
C) Dry Period Categories
  Based to the values of Table 4, It is well known that both shortening and complete omission of the DP reduces milk production in the subsequent lactations; continuous milking results in significant production losses (Steeneveld et al. 2013; Andersen et al. 2005; Klusmeyer et al. 2009; Mantovani et al. 2010). When shortening the dry Period length, a decrease in milk production is often reported, but this decrease is not always significant (Gulay et al. 2003; Pezeshki et al. 2007; Santschi et al. 2011).
  It is interesting to note that the highest milk yield (32.28 kg) that achieved with 60–90 days of dry before calving was associated with the highest AFR (1.74 kg min^−1^) and milk flow rates during all studied milking intervals as follows: 0.29, 1.43, 1.34, and 2.00 kg min^−1^ for 0:15, 15:30, 30:60, and 60:120 s of milking, respectively. These results demonstrate that the ideal dry-period before calving is 60–90 days in terms of daily milk yield and milk flow rate.
  These results agreement with literature that ensuring a dry-period (DP) of 61:80-days will translate into increased cows performance, as well as streamlining the financial cycles of the farms; where they noticed a significant influencing of milk yield by the 61:80-days DP, whilst a DP ≤ 40-days or ≥ 80-days induced losses in milk yield (Radu et al. 2021).
  In Turkish herd of Holstien cattle, dry-period length (DPL) was classified in five categories (≤ 40, 41:50, 51:60, 61:70, and ≥ 71-day); The differences in 305-dMY of DPL groups were non-significant. The highest 305-dMY (7808.6 ± 135.1 Lt) was obtained from ≥ 71 day -group and the lowest 305-dMY (7529.4 ± 159.8 Lt) was obtained from ≤ 40 day-group and There was a positive correlation tend to 0.056 between DPL, and 305-dMY (Kiyici et al. 2020).
D) Times of Mastitis Infection
  Bovine mastitis, an inflammation of the mammary gland, is the most common disease of dairy cattle causing economic losses due to reduced yield and poor quality of milk (Cheng and Han 2020) and (Gomes and Henriques 2016). Also, mastitis cows often never recovered their potential yield (Gröhn et al. 2004).
  Based to the values of Table 5, The highest peak milk flow rate was recorded with non-infected animals (3.65 kg min^−1^) and the lowest (3.26 kg min^−1^) was for infected animals' ≥ 4-times, whereas were 3.63, 3.48, and 3.53 kg min^−1^ for infected animals 1, 2, and 3-times, respectively. In contrast to milk yield and AFR, there was a significant increase in milking duration by increase mastitis infection times, which were 4.1, 4.26, 4.34, 4.29, and 4.51 min of infected animals 0, 1, 2, 3, and ≥ 4-times, respectively.
  Milk flow rates during 0:15 s of milking isn’t affected by mastitis infection times. The milk flow rates during the rest of studied milking intervals did not show a clear trend, as the highest values were at 15:30 s of milking with animals that were infected 3 times (1.46 kg min^−1^) and the lowest in animals infected ≥ 4 times (1.33 kg min^−1^). The highest values were at 30:60 s of milking with animals that were infected 3 times (1.37 kg min^−1^) and the lowest with non- infected animals (1.24 kg min^−1^). On the other hand, the highest values were at 60:120 s of milking with non- infected animals (1.99 kg min^−1^) and the lowest in animals that were infected ≥ 4-times (1.73 kg min^−1^). These results are contrary to the results of AFR, which had had a clear trend; this may be due to the failure to record the rate of milk production during the rest of milking intervals. Studies published in this area are relatively few and require additional specialized studies.

### The curve estimation for Milking-abilities all over lactation period

Based to the values of Table 6, The LSMs of milk yield by various milking frequency and interval treatment for early-lactation (24-DIM) were 22.91, 18.12, 21.46, 20.86, and 20.75 ± 0.364 kg day^−1^, mid-lactation (136-DIM) were 19.16, 16.65, 18.89, 18.40, and 18.58 ± 0.208 kg day^−1^, with milking-time 10.94, 7.19, 9.29, 9.16, and 9.13 ± 0.215 min day^−1^ for early-lactation and 9.4, 6.77, 8.41, 8.62, and 8.23 ± 0.197 min day^−1^ for mid-lactation, for twice a day, once a day; and 3 regimes of milking 3 times in 2 days: 12–18-18 h, 10–19-19 h, and 8–20-20 h (Hall 2023). During the lactation, the mammary growth stops after the peak of lactation (about 6 weeks after calving), so the "regression of the mammary gland" begins, and this continues even after the end of lactation. Mammary regression is the result of a combination of a decrease in the number of secretory cells and the secretion rate of those that remain active. Histological changes during this period in dairy cows are characterized by a decrease in the number of milk-secreting cells per alveolus, a reduction in the size and number of the mammary alveoli, and consequently a gradual decrease in milk production (Dahl 2020).

These results show that all cubic models have a better predictive ability of milking-abilities criteria through the time curve parameters than the linear models. Non-linear regression is more flexible and accurate for lactation curve fitting than linear regression (Motulsky and Christopoulos 2004).

Regression splines (quadratic and cubic spline models with three knots) showed better fitting performances and greater flexibility for all milk traits. In addition, estimated determination coefficients (R^2^) for various lactation curve models ranged between 0.94 to 0.97 for Tunisian Holstein–Friesian cows (Bouallegue et al. 2015). The algebraic model fitted by a nonlinear regression procedure to the data resulted in reasonable prediction curves for milk yield (R^2^a of 0.89) (Santos and Silvestre 2008). Correlation between predicted and observed milk yields was highest in the polynomial model at 0.997, lowest in the Pollott model (0.764), and averaged 0.989 for all the models (Adediran et al. 2012).

Lactation functions provide an elegant example to study the applicability of mathematical models to explain nonlinear phenomena in animal science (Oliveira et al. 2020). The adjustment of the lactation curve has traditionally been performed using mathematical models through linear and non-linear regression. However, these analytical tools have several limitations, mainly related to the non-linear pattern of the lactation curve. (Guevara et al. 2023).

In conclusion, milking-ability criteria are influenced by a multitude of managerial factors, including times of milking, herds’ men (milking team), dry-period, udder health (mastitis infection). dairy farmers can enhance milking efficiency and achieve maximum milk production from their cattle via taking main managerial factors affecting milking-abilities in focus and enhance it. The curve of milk yield and milking abilities were inconstant all over lactation period, that must be considered. In this context, further studies should be conducted to study the effect of managerial factors on the quality of produced milk, such as its content of protein, fat, and mineral elements, in addition to the total bacterial and somatic cell count in the milk.

## Data Availability

Data will be made available on request.
